# The role of platelets in the blood-brain barrier during brain pathology

**DOI:** 10.3389/fncel.2023.1298314

**Published:** 2024-01-08

**Authors:** Weifeng Lv, Xiaofan Jiang, Yanyu Zhang

**Affiliations:** Department of Neurosurgery, Xijing Hospital, Fourth Military Medical University, Xi’an, China

**Keywords:** BBB integrity, platelet-released factors, neuroinflammation, thrombosis, PDGF

## Abstract

Platelets play critical roles in maintaining hemostasis. The blood brain barrier (BBB), a significant physical and metabolic barrier, helps maintain physiological stability by limiting transportations between the blood and neural tissues. When the brain undergoes inflammation, tumor, trauma, or bleeding, the platelet responses to help with maintaining BBB homeostasis. In the traditional point of view, activated platelets aggregate to form thrombi which cover the gaps of the blood vessels to protect BBB. However, increasing evidences indicate that platelets may harm BBB by enhancing vascular permeability. Hereby, we reviewed recently published articles with a special focus on the platelet-mediated damage of BBB. Factors released by platelets can induce BBB permeability, which involve platelet-activating factors (PAF), P-selectin, ADP, platelet-derived growth factors (PDGF) superfamily proteins, especially PDGF-AA and PDGF-CC, etc. Platelets can also secrete Amyloid-β (Aβ), which triggers neuroinflammation and downregulates the expression of tight junction molecules such as claudin-5 to damage BBB. Additionally, platelets can form aggregates with neutrophils to release reactive oxygen species (ROS), which can destroy the DNA, proteins, and lipids of endothelial cells (ECs). Moreover, platelets participate in neuroinflammation to affect BBB. Conversely, some of the platelet released factors such as PDGF-BB, protects BBB. In summary, platelets play dual roles in BBB integrity and the related mechanisms are reviewed.

## 1 Introduction

Blood brain barrier (BBB) is the special feature of the vasculature in the central nervous system (CNS), which is functioning as a physical and metabolic barrier to limit transportation between the blood and neural tissues to maintain brain homeostasis, regulate influx and efflux transportation to protect against harmful agents or microorganisms in the blood (Arvanitis et al., 2020). The neurovascular unit of brain vessels consist of ECs, basal lamina and the surrounding cell structure such as pericyte and astrocytic endfeet. In the brain ECs, the vascular permeability related molecules such as tight junction molecules and transporters are differently expressed, compared to the vascular ECs of the other organs, which is one of the characteristics of BBB (Xie et al., 2019). Recently published articles indicate that disturbance of BBB is widely occurred during brain pathology, such as Parkinson’s disease, ischemia stroke, and Alzheimer’s disease (AD) (Wisniewski and Kozlowski, 1982; Chung et al., 2016; Song et al., 2021). Therefore, exploring the mechanism of BBB damage under brain pathology and seeking for therapeutic targets may provide new insights for improving prognosis of patients.

Platelets, the smallest blood cells, are anucleated and originated from the hematopoietic lineage of the megakaryocyte. The amount of platelets in healthy individuals are around 100–150 × 10^9^/L in the blood (Vinholt, 2019). Megakaryocytes produce platelets both from the bone marrow and other tissues, especially the lung (Lefrançais et al., 2017). After production, platelets are released into the blood flow to maintain hemostasis (Davì and Patrono, 2007). The primary function of platelets is thrombosis, which mechanism has been widely explored (Yeung et al., 2018). Thrombus formation plays critical roles in initiation and progression of many diseases, including chronic thromboembolic pulmonary hypertension (Hsin and Fan, 2021), acute myocardial infarction (Keeley and Hillis, 1996), intracerebral hemorrhage (ICH) (Weimar and Kleine-Borgmann, 2017), and so on. A series of antiplatelet drugs are available for clinical use. For example, both aspirin and P2Y12 inhibitors are prescribed to patients with traumatic brain injury (TBI) (Choi et al., 2017). In the opposite way, platelet transfusion is also used as a therapeutic method to cure a series of brain disease since the formation of thrombus can cover the injury and trigger the recovery process. For instance, Miles et al. (2022) found that platelet transfusion may prevent patients with TBI from neurosurgical interventions. Platelet transfusion is regarded as a traditional therapeutic strategy for brain diseases, especially in ICH, which is the second leading cause of stroke-related death and disability (Stanworth and Shah, 2022). The relationship between platelets and ICH inspired researchers to explore the interaction between platelets and the BBB (Luo et al., 2019; Stanworth and Shah, 2022). Luo et al. (2019) transfused platelets into the mice with collagenase induced ICH or transient middle cerebral artery occlusion. They found that platelet transfusion safeguard BBB integrity and suggested that transfusion of resting platelets may be useful to improve the safety of tissue plasminogen activator (tPA) based thrombolysis in ischemic stroke (Luo et al., 2019). In addition, antibody blockade to platelet glycoprotein (GP) Ib reduces platelet activation and the formation of thrombus in ischemia stroke, which improves the BBB integrity (Chen et al., 2018). Furthermore, PAF was shown to induce transient opening of the BBB to facilitate brain penetration of edaravone (Fang et al., 2014), which implied that platelets can enhance BBB permeability.

In the nervous system, platelets were reported to play critical roles in initiation and progression of many diseases of the brain, such as neuroinflammation (Anderson et al., 2019), AD (Beura et al., 2022), glioma (Haemmerle et al., 2018), and ICH (Weimar and Kleine-Borgmann, 2017). For instance, it is reported that platelets aggregate with neutrophils to release ROS and inflammatory factors to stimulate brain cell injury and neuroinflammation in TBI (Hubbard et al., 2021; Kempuraj et al., 2021). Besides, impaired platelet-derived growth factors signaling pathway (such as disturbance of PDGF-BB/PDGFRβ or activation of latent PDGF-CC/PDGFRα) (Su et al., 2015; To et al., 2023) can also increase BBB permeability in TBI patients. In addition, Paul et al. (2015) proposed that disturbance of PDGF-BB/PDGFRβ signaling pathway by administration of rhPDGF-BB is a key link in promoting prognosis in patients with Parkinson’s disease. Thrombosis and vasoconstriction mediated by platelets were identified to lead secondary injury in Parkinson’s disease (Lin, 2013). Moreover, disruption of the BBB is a major pathological change during ICH, which is a result from oxidative stress and inflammation (Chen et al., 2022). BBB damage is one of the critical mechanisms of ICH-induced brain injury and is closely associated with poor prognosis (Xi et al., 2017). Platelet transfusion as a traditional therapeutic approach for ICH, inspired researchers to explore the relationship between platelets and the BBB (Luo et al., 2019; Stanworth and Shah, 2022). Interestingly, several studies implied that activation and aggregation of platelets disturbes the function of BBB and enhances vascular permeability. Besides what has been mentioned above, platelet-mediated pathological changes have also been found in a series of brain pathological diseases, which are summarized as follows (Table 1). The interactions between platelet related signaling and the BBB of brain diseases need to be further investigated, which may provide a new angel to understand the initiation and progression of the brain diseases and may offer new approaches for patients.

**TABLE 1:** Dysfunction of Platelet related signaling on BBB in Brain Diseases.

Diseases	Mechanism	References
TBI	1. PDGF-BB/PDGFRβ signaling impairment results in the loss of ECs interactions	Bhowmick et al., 2019; To et al., 2023
	2. Platelets and neutrophils form aggregates and release ROS	Hubbard et al., 2021
	3. Platelets release inflammatory factors to stimulate neuroinflammation	Kempuraj et al., 2021
	4. Platelets can activate latent PDGF-CC in the brain to promote BBB permeability through PDGFRα	Su et al., 2015
Multiple sclerosis	1. PDGF-BB/PDGFRβ signaling impairment results in the loss of EC interactions	Scalise et al., 2021
	2. Platelets activate a series of inflammatory responses	Callea et al., 1999
	3. To activate latent PDGF-CC in the brain to promote BBB permeability through PDGFRα	Lewandowski et al., 2016
	4. Activate the expression of PDGF-AA and result in the loss of ECs adhesion molecules through PDGFRα	Girolamo et al., 2019
	5. Activate the expression of PAF and trigger inflammation	Callea et al., 1999
	6. Promote the transmigration of human CD4 + T-cell subsets by PECAM-1	Wimmer et al., 2019
	7. Downregulate the expression of PECAM-1 which functions as an adhesion molecule	Souza et al., 2017
Diabetic encephalopathy	1. Platelets activate a series of inflammatory responses	Rom et al., 2019
	2. Suppress the expression of PDGF-BB which results in the loss of ECs interaction	Li et al., 2021
Japanese encephalitis	Mediate the adhesion and infection of Japanese encephalitis virus (JEV) by PAF	Ashraf et al., 2021
Parkinson’s disease	1. PDGF-BB/PDGFRβ signaling impairment results in the loss of EC interactions	Paul et al., 2015
	2. Activated platelets lead to secondary injury via thrombosis and vasoconstriction	Lin, 2013
AD	1. PDGF-BB/PDGFRβ signaling impairment results in loss of EC interactions	Nation et al., 2019
	2. To activate latent PDGF-CC in the brain to promote BBB permeability through PDGFRα	Scalise et al., 2021
	3. Increase circulating beta-amyloid to trigger neuroinflammation by downregulating tight junction molecules and promotes ROS production	Carrano et al., 2011; Nation et al., 2019
	4. Downregulate the expression of PECAM-1 which functions as an adhesion molecule	Williams et al., 2022
	5. Induce Ca^2+^ dyshomeostasis which might be mediated by the PAF formation	Miners et al., 2018
Glioblastoma	PDGF-BB/PDGFRβ signaling impairment results in the loss of EC interactions	Mittapalli et al., 2016
Stroke	1. To activate latent PDGF-AA in the brain to promote BBB permeability through PDGFRα	Nguyen et al., 2021
	2. PDGF-BB/PDGFRβ signaling impairment results in the loss of ECs interactions	Guo et al., 2021
	3. To activate latent PDGF-CC in the brain to promote BBB permeability through PDGFRα	He et al., 2023
	4. Platelets and neutrophils form aggregates and release ROS	Ye et al., 2022
	5. Platelets active neuroinflammation	Sun and Langer, 2022
	6. Form PNA and release ROS	Rodrigues and Granger, 2015
	7. Promote adhesion of neutrophils to peripheral ECs	Arnould et al., 1993
ICH	Induce Ca^2+^ dyshomeostasis which might be mediated by the PAF formation	Fang et al., 2011

## 2 Platelet-released factors or platelets complexes jeopardize BBB during brain pathology

The platelet and its released factors are involved in different brain pathologies as mentioned above, which affect BBB and disease progression. In this article, we reviewed the typical platelet-released molecules and platelets complexes of their effects on BBB and summarized the mechanisms (Table 2).

**TABLE 2:** The Mechanism of Platelets in Compromising BBB Integrity.

Platelet derived bioactive factors	Mechanism	Reference
PDGF	1. Disturbed PDGF superfamily expression	Nation et al., 2019; Scalise et al., 2021; Li et al., 2022; He et al., 2023
	2. Impairment of PDGFRβ signaling pathway or activation of PDGFRα signaling pathway	Bhowmick et al., 2019; Nguyen et al., 2021
PNA	1. Release ROS	van Wetering et al., 2002; Boueiz and Hassoun, 2009
	2. Release P-selectin and sCD40L	Vital et al., 2010
PAF	1. Regulation of tight junction-associated protein	Brailoiu et al., 2018
	2. Affect BBB permeability synergistically with P-selectin	Snyder et al., 2015
	3. Promote the interaction between ECs and inflammatory cells	Benveniste et al., 1972; Arnould et al., 1993; Park et al., 1999
	4. Compromising BBB by regulating tight junctions and the morphology of ECs directly	Fang et al., 2011
Aβ	1. Trigger a series of neuroinflammation	Carrano et al., 2011
	2. Compromising BBB directly by changing the ability of EC transportation of nutrients, vitamins, etc.	Zlokovic, 1997; Jancsó et al., 1998
	3. Impairment of PDGFRβ signaling pathway or activation of PDGFRα signaling pathway	Miners et al., 2018
	4. Promote Ca2 + influx	Shi et al., 2010
Neuroinflammatory factors	1. Release inflammatory factors such as TNF-α, IL-1β and IL-10	Reed et al., 2000; Rainesalo et al., 2005; Ojha et al., 2019; Cicognola et al., 2023
	2. Affect BBB permeability synergistically with platelet factor 4 (PF4), platelet-derived growth factor β (PDGFβ) and sCD40L	Jones et al., 2016
GP Ib	Downregulate tight junction proteins; promote F-actin cytoskeleton rearrangement	Chu et al., 2021
Adenosine diphosphate (ADP)	Stimulate platelet activation to release PAF, sCD40L and P-selectin	Zolotoff et al., 2021

### 2.1 PDGFs

Platelet-derived growth factors (PDGF) superfamily contains five functional growth factors: PDGF-AA, PDGF-BB, PDGF-AB, PDGF-CC, and PDGF-DD. These proteins consist of four polypeptide chains denoted as PDGF-AA to -DD, and are located on the chromosome 7, 22, 4, and 11 in humans (Fredriksson et al., 2004). PDGFs are broadly expressed in multiple tissues and are secreted by ECs, macrophages, and epithelial cells. The other main source of PDGFs are platelets, which release PDGFs upon degranulation (Papadopoulos and Lennartsson, 2018). Their receptors are classified into PDGFRα and PDGFRβ. Polypeptide chains PDGF-AA and PDGF-CC binds to the PDGFRα, whereas PDGF-BB and PDGF-DD binds to the PDGFRβ. PDGFs play critical roles in embryonic development, angiogenesis, kidney development, and tumorigenesis (Andrae et al., 2008; Cao, 2013; Demoulin and Essaghir, 2014; Ehnman and Östman, 2014).

Disturbed PDGF ligands or receptors result in malignance such as hematopoietic, glial, and soft-tissue cancers and non-cancerous disorders such as skeletal defects, brain calcification, and vascular anomalies (Guérit et al., 2021; Lv et al., 2022). Up to now, investigations regarding to the relationship between PDGF and BBB are mostly focused on ICH, as well as diabetic encephalopathy (Rom et al., 2020), Parkinson’s disease (Bondarenko and Saarma, 2021), multiple sclerosis (Cao et al., 2022), GBM (Mittapalli et al., 2016) and AIDS encephalopathy (Nguyen et al., 2021). A study reported that disruption of BBB is correlated with downregulation of PDGFRα, indicating PDGFRα maintains the BBB integrity by using the middle cerebral arterial occlusion (MCAO) model in mice (Nguyen et al., 2021). The mechanisms of PDGF-mediated changes of the BBB can be classified into two aspects: (1) disturbed PDGF superfamily proportion and (2) altered PDGFR signaling pathways including impairment of PDGFRβ or activation of PDGFRα in targeted ECs. Recently published articles suggest that each member of the PDGF superfamily plays a distinct function in BBB homeostasis. PDGF-AA and PDGF-CC may damage the BBB integrity whereas PDGF-BB protects it. The expression level of PDGF-BB can be used as an indicator of BBB integrity (Nation et al., 2019; Scalise et al., 2021). For instance, He et al. (2023) established a rat thromboembolic stroke model with disrupted BBB to explore the relationship between delayed admission of recombinant tissue plasminogen activator (rtPA, alteplase) and hemorrhagic transformation (HT). They assessed the brain infarction, neurological outcomes, BBB permeability, and ICH in the rodent model of thromboembolic stroke by rtPA thrombolysis with or without remote ischemic conditioning (RIC). They found RIC protects BBB against HT injury, and the expression of PDGF-CC is negatively correlated with RIC-mediated protection of BBB. Additionally, the protective effect of RIC can be blocked by intravenous or intraventricular supplementation with recombinant PDGF-CC, which indicates that PDGF-CC enhances permeability of the BBB and causes a secondary injury (He et al., 2023). In Parkinson’s disease, PDGFs and their corresponding receptor pathways were confirmed to participate in the disturbance of BBB via regulating mitochondrial function, oxidative stress, Ca^2+^ homeostasis, protein misfolding and aggregation, neuroinflammation and peripheral astrocytes and macrophages (Li et al., 2022). The second mechanism of PDGF-mediated changes of the BBB involves the impairment of the PDGFRβ signaling or activation of the PDGFRα signaling pathways in the targeted ECs. Bhowmick et al. (2019) established a fluid percussion injury (FPI) model in mice and examined the relationship between the expression of various pericyte markers (including PDGFR-β, NG2, and CD13) and the gene expression related to BBB permeability. They found that TBI causes disturbance of the PDGF-BB signaling pathway in ECs, which lead to decreased expression of tight junction molecules between ECs and other cells such as pericytes and astrocytes (Bhowmick et al., 2019). However, in contrary to the previous theories, PDGFRα was shown to participate in compromising the integrity of BBB. Nguyen et al. (2021) remodeled mice with MCAO, and found that as soon as MCAO occurs, the expression of PDGFRα in ECs was downregulated and is mediated by TGF-β1. The infraction lesion was enlarged by downregulation of the PDGFRα pathway, indicating PDGFRα protects BBB. Paradoxically, PDGF-AA and PDGF-CC which was traditionally found to damage BBB, can, however, bind to PDGFRα which was reported to protect BBB. Therefore, it assumes that PDGFRα could be activated atypically in much more comprehensive mechanisms. The function of PDGFs in maintaining BBB needs to be deeper investigated.

### 2.2 PNAs

After activation, platelets are prone to form aggregates with lymphocytes such as neutrophils, to play critical roles in secondary BBB injury (Rodrigues and Granger, 2015). In pathological conditions, such as injury or inflammation, PNAs are formed and recruited into the lesions to stimulate neuroinflammation and BBB dysfunction. These aggregates (PNAs) promote the release of several bioactivators, such as ROS, P-selectin, and sCD40L, to regulate BBB integrity.

Reactive oxygen species (ROS), such as hydrogen peroxide (H_2_O_2_) and singlet oxygen (O_2_), encompasses oxygen free radicals such as superoxide anion radical (O2^–^), hydroxyl radical (OH), and non-radical oxidants, which can interconvert to be each other (Zorov et al., 2014). ROS are generated in the situations of decreased free radical scavenging enzyme, increased glucose metabolism, or increased presence of growth factors and cytokines. Disturbance of mitochondria, NADPH oxidases (NOX), cyclooxygenases (COX), lipoxygenases, xanthine oxidases, or cytochrome P450 enzymes also contributes to ROS formation (Moloney and Cotter, 2018). DNA, proteins, and lipids are the targets of ROS which results in genetic instability and cell death (Liou and Storz, 2010; Stanicka et al., 2015). ROS can also affect transcription through regulating transcription factors such as hypoxia-inducible factor-1 (HIF-1), which triggers the expression of several genes including VEGF (Plastino et al., 2021). By ROS exposure, ECs experience disruptions of the inter-endothelial junctions, actomyosin contraction, gap formation, and altered expression and phosphorylation of junctional adhesion molecules, which together promote BBB permeability (van Wetering et al., 2002; Boueiz and Hassoun, 2009). However, the function of PNA in BBB damage in brain pathology was mostly reported in TBI, which needs to be investigated in other pathological conditions.

P-selectin, a pre-synthesized protein stored in the platelet α-granules, also named as CD62P, is belonging to the CD62 family (Furie and Furie, 1995; Hartwell and Wagner, 1999). P-selectin mediates the adhesion of platelets with other cells such as neutrophils (Qi et al., 2015), and triggers quick exposure of tissue factors to regulate hemostasis (Ivanov et al., 2019). P-selection plays a critical role in platelet-mediated injury of BBB. Firstly, P-selectin mediates platelet adhesion to leukocytes. After activation, platelets form heterotypic aggregates with leukocytes by P-selectin. PNAs produce more than twice the amount of superoxide than their free counterparts, and release almost twice as much PAF as when the cells were activated separately (Nagata et al., 1993; Park et al., 1999). Secondly, P-selectin can also compromise BBB independently. In a study by Kisucka et al. (2009), P-selectin overexpress P-selectin mice showed higher BBB permeability and atherosclerotic lesions, which resulted in a higher risk of brain infarction. This is a direct evidence showing that free P-selectin in plasma can promote BBB permeability (Kisucka et al., 2009). Similarly, Vital et al. (2010) implanted angiotensin II (AngII)-loaded osmotic pumps in mice, by which they revealed that AngII injection can upregulate P-selectin and sCD40L in blood, which in turn increases BBB permeability indicated by increased Evans blue leakage. In summary, PNA formation plays a critical role in platelet-mediated BBB injury, involving oxidant stress and the release of bioactive factors. However, the molecular mechanism of P-selectin on BBB permeability, as well as whether P-selectin is involved in the PNA-derived oxidant stress, or whether there is a synergistic effect between the P-selectin and PNA, is still unclear.

### 2.3 PAF

Platelet-activating factors (PAF) was once thought to be produced only by ECs. However, a recently published article on the obstructive sleep apnea claimed that activated platelets can release PAF to retain platelet aggregation (Zolotoff et al., 2021). PAF is a bioactive phospholipid messenger. The primary function of PAF is to activate platelets to induce platelet aggregation and inflammation (Stanimirovic and Satoh, 2000), which triggers a series of physiological or pathological processes such as rheumatoid arthritis (Hilliquin et al., 1995) and vascular permeability (Jeewandara et al., 2015).

Platelet-activating factors (PAF) can influence BBB integrity (Zimmerman et al., 1990). According to the recently published articles, PAF mediates neuroinflammation during both the initiation and progression of brain diseases including Japanese encephalitis (Ashraf et al., 2021), multiple sclerosis (Callea et al., 1999) and aseptic inflammatory phase of cerebral hemorrhage (Frerichs et al., 1990), among which ICH is mostly reported. This indicates that PAF can act as a neuroinflammation mediating factor to jeopardize BBB. In an *in vitro* study, after PAF treatment, ECs developed leakier BBB (Fang et al., 2011). *In vivo*, rats infused with PAF intravenously showed increased Evans blue leakage and mild edema formation. In addition, the concentrations of edaravone, an antioxidant, was also increased in tissue interstitial fluid which indicated enhanced edaravone penetration into the brain. It suggests that PAF induces transient BBB opening which allows the penetration of neuroprotective edaravone (Fang et al., 2014). The underlying mechanisms can be as follows: (1) PAF activates actin polymerization and Ras-related C3 botulinum toxin substrate 1 (Rac1)-dependent junctional protein relocation, both of which incorporate into the membrane phospholipid bilayer and exert their activities on cell membranes (Frerichs et al., 1990; Rodrigues and Granger, 2015). Brailoiu et al. (2018) investigated the effect of PAF on the expression of tight junction molecules. They found that PAF treatment on the rat brain microvessel endothelial cells (RBMECs) decreased the expression of ZO-1, a tight junction-associated protein, which indicated that PAF can bind to receptors on ECs to influence the tight junction molecules. Moreover, PAF treatment resulted in intercellular gaps of ECs to disrupt endothelial barrier function by using electric cell-substrate impedance sensing (ECIS), which indicates compromised BBB (Brailoiu et al., 2018). (2) Synergistic effects of PAF with other molecules that affect BBB integrity. PAF production is reported to be associated with P-selectin expression. For instance, agonists targeting PAF production also affect P-selectin expression. P-selectin is indispensable for PAF to activate leukocytes (Klionsky et al., 2016). (3) PAF promotes the interplay between ECs and inflammatory cells. PAF is produced/secreted by several cell types and stored in platelets. PAF was initially discovered as a substance which is responsible for platelet aggregation (Benveniste et al., 1972). In patients with ischemic stroke, neutrophils were shown to adhere to peripheral ECs in a PAF-dependent manner (Arnould et al., 1993). The leukotrienes LTC4 and LTD4 are PAF metabolites, which stimulate ECs to produce PAF and bind to neutrophils in a positive feedback loop. LTC4 and LTD4 can mediate the binding between neutrophils and ECs and close the loop of arachidonic acid metabolites-meditated augmentation of inflammatory actions. Park et al. (1999) also reported similar findings in newborn piglets. They administered newborn piglets with PAF and explored the relationship between PAF and BBB integrity by using intravital epifluorescence videomicroscopy, closed cranial windows, and leukocytes labeling by rhodamine 6G. They reported either exogenous PAF or asphyxia-reoxygenation can result in a loss of PNAs which compromise BBB integrity. This finding indicates that PAF contributes to leukocyte adherence and BBB breakdown in cerebral ischemia (Park et al., 1999). (4) PAF compromises BBB directly. Fang et al. (2011) reported that PAF treatment on RBMECs resulted in reduced survival rate and leakier BBB. This is consistent with the previous study by Brailoiu et al. (2018).

In summary, PAF influences BBB integrity through platelet activation, regulation of tight junctions molecules, disrupting the morphology of ECs, and affecting the expression of other factors such as P-selectin. PAF could be a therapeutic target in preventing secondary BBB injury in patients with TBI and inflammation, and the mechanisms need to be further investigated.

### 2.4 Aβ

It is increasingly recognized that the disruption of blood vessels contributes to cognitive impairment (Snyder et al., 2015; Gottesman et al., 2017). The increased permeability of BBB ensures the infiltration and accumulation of beta-amyloid, a leading cause of AD (Halliday et al., 2000). Platelets are sources of beta-amyloid. Enhanced platelet activation and increased circulating beta-amyloid have been identified in AD (Halliday et al., 2000).

Aβ and pTau accumulation are the leading causes of AD, which trigger vasoactive and/or vasculotoxic effects (Guérit et al., 2021). The formed amyloid plaques wrap vessels and break down the BBB, which has been shown histologically by postmortem examination on human AD brains (Wisniewski et al., 1997). The mechanism is that Aβ as a toxic protein triggers neuroinflammation which results in downregulation of tight junction molecules between ECs. The expression of claudin-5 and other tight junction molecules are significantly suppressed in the capillaries of AD patients. In addition, Aβ secreted by platelets can promote the activity of nicotinamide adenine dinucleotide phosphate oxidase 2, an enzyme in microglia, which plays a critical role in ROS production in damaged capillaries (Carrano et al., 2011). In rat AD models, the researchers infused Aβ1–42 or Aβ1–40 into rats by intracarotid injection to increase BBB permeability and they found that the infusion increased BBB transportation of nutrients, vitamins, and receptor and/or carrier-mediated transportation of peptides, proteins, certain viruses and leucocytes (Zlokovic, 1997; Jancsó et al., 1998). These findings indicate that beta-amyloid damages ECs which are the essential component of BBB. Moreover, increased BBB permeability, cerebral vasoconstriction, and increased platelet aggregation can predispose to thrombotic and/or ischaemic events in the AD brains, which again triggers platelets to release Aβ. In AD, by examining the white matters from 49 patients and 37 control brains, researchers found that the accumulation of Aβ was usually accompanied by the downregulated PDGF-BB. Consistently, by checking the fibrinogen leakage, they found decreased BBB integrity in AD. Furthermore, loss of PDGF-BB within the precuneus in AD was found to be associated with fibrinogen leakage, increased oxygenation, and fibrillar Aβ accumulation. These findings indicate that Aβ is negatively correlated with BBB integrity (Miners et al., 2018). Last but not least, It was reported that oligomeric Aβ1-42, a member of the amyloid beta peptide family, can bind to N-methyl-D-aspartic acid (NMDA) receptors in ECs, and then activates protein kinase C (PKC) to induce Ca^2+^ influx, which is one of the indicators of BBB integrity. Additionally, the Ca^2+^ influx mediated by Aβ1-42 via NMDA/PKC can be blocked by the Ginkgo biloba extract EGb761, a free radical scavenger with PAF antagonism. This indicates that Aβ1-42-induced Ca^2+^ dyshomeostasis might be mediated by PAF (Shi et al., 2010). In summary, Aβ accumulation is the hallmark of AD, which can be released by platelets. Aβ compromises BBB by regulating tight junction molecules through PDGF, PAF, or other bioactive molecules.

### 2.5 Neuroinflammatory factors

Neuroinflammation nearly participates in all kinds of neurological diseases in brain (Adzemovic et al., 2013). Neuroinflammation and BBB disruption have been identified as two critical mechanisms of ischemia stroke-induced brain injury (Zhu et al., 2018). Neuroinflammation in ischemia stroke mediates the first entry of leukocytes especially neutrophils (Drieu et al., 2018). Both anti-platelet strategies (Weiland et al., 2021) and platelet transfusion (Luo et al., 2019) have been applied to target neuroinflammation in ischemic stroke. According to a study conducted by Luo et al. (2019) platelet transfusion can significantly reduce hematoma volume after ICH and attenuates the tPA-induced brain hemorrhage. In opposite, other studies indicate that platelets are stimulators of neuroinflammation by releasing TNF-α, IL-1β and IL-10 (Heofilis et al., 2021). In addition, platelet granules contain key neurotransmitters, such as dopamine, glutamate, histamine, serotonin, and ATP, which can influence the pathological processes of neurodegenerative diseases (Reed et al., 2000; Jones et al., 2016; Ojha et al., 2019; Cicognola et al., 2023). Moreover, platelet-derived cytokines play critical roles in regulating neuroinflammation and viral infection. For example, platelets were reported to participate in JEV-mediated neuroinflammation. PF4, also known as CXCL4, was found to mediate the proliferation of JEV via PF4-CXCR3-IFN axis (Chu et al., 2021). Soluble PDGFRβ, released by platelets, destroys BBB integrity. Cicognola et al. (2023) examined the data from 771 participants with different degrees of cognitive impairment and inspected the relationship between the cerebrospinal fluid PDGFRβ levels and AD-associated pathologic changes. Higher cerebrospinal fluid PDGFRβ levels were found to be positively related to the age and the degree of neuroinflammation. Since soluble PDGFRs can be released by platelets, it is reasonable to suggest that platelets can regulate neuroinflammation by PDGFRs. Last but not least, sCD40L is another important molecule released by platelets. Jones et al. (2016) established an HIV-associated neurocognitive disorder (HAND) model in mice by infecting wild-type (WT) mice with EcoHIV. Two weeks post-infection, increased permeability of BBB was detected. The expression level of the tight junction molecule claudin-5 was decreased due to platelet activation and enhanced secretion of sCD40L (Fredriksson et al., 2004). To summarize, platelets play contradictory functions on BBB integrity.

### 2.6 Other platelet-derived factors that influence BBB integrity

There are also other platelet-released molecules which were reported to affect BBB integrity but with only limited evidences. Firstly, GP Ib as a platelet receptor is existed on the platelet membrane. Anfibatide (ANF), a GP Ib antagonist was reported to significantly alleviate BBB disruption in mice with cerebral ischemia/reperfusion (CI/R) injury (Chu et al., 2021). GP Ib senses the injury in blood vessels and the platelet was activated via the Epac signaling pathway. The activated platelets then downregulated the expression of tight junction proteins and promoted F-actin cytoskeleton rearrangement to increase the permeability of BBB in the ischemic brain tissue, which triggers brain edema. Secondly, platelet-derived ADP, an important factor contributing to platelet aggregation, was reported to be negatively associated with BBB integrity by stimulating PAF, sCD40L and P-selectin releasement (Demoulin and Essaghir, 2014). However, the mechanisms of both that were mentioned above require further investigations.

## 3 Conclusion

**FIGURE 1:**
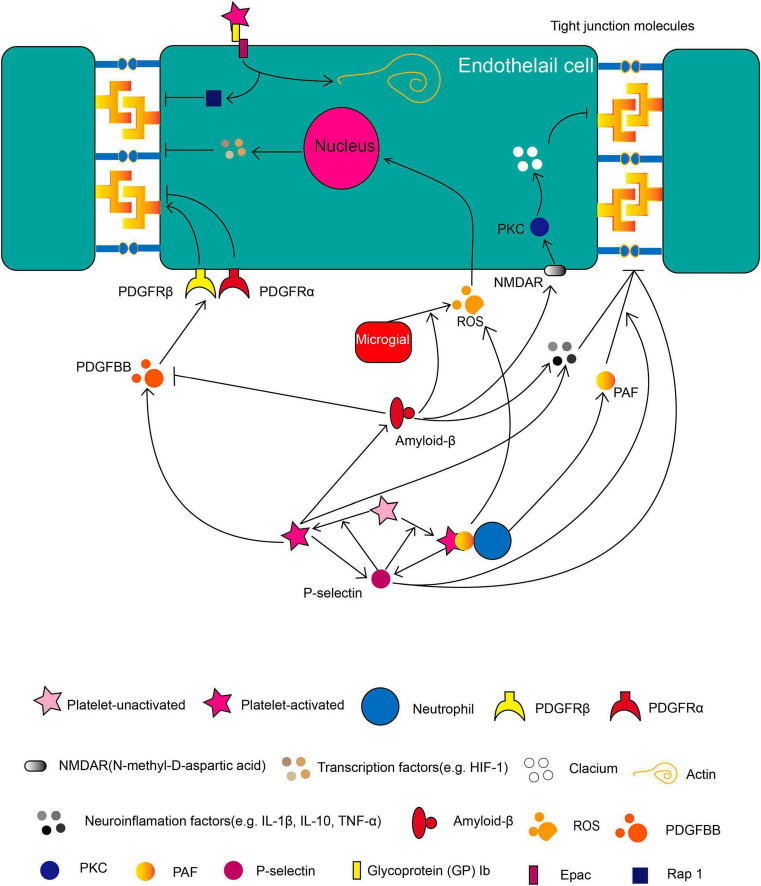
The role of platelets in BBB during brain pathology. Molecules released by activated platelets such as soluble P-selectin, PDGF, Aβ, PAF, various neuroinflammatory factors and the membrane-binding protein such as GP 1b regulates BBB integrity in different pathways as illustrated. Besides, platelets can form PNA (platelets-neutrophil aggregate) to affect BBB integrity. The directional arrow indicates the effect of promoting. The suppressive arrow indicates the effect of inhibiting.

Platelets, which are activated by vascular insult or injury, play a critical role in regulating thrombus formation and maintaining the hemostasis of blood vessels. The function of platelet aggregation is to form clots to cover the gap in the lesions. In addition to their classic roles, recently published articles indicated that platelets also play important roles in neuroinflammation and BBB integrity. In this paper, we reviewed articles that explored the factors from platelets in relation to BBB integrity (Figure 1). Especially, the PDGF superfamily members, PAF, PNA, Aβ, and neuroinflammation factors such as TNF-α, IL-1β, and IL-10 play crucial roles in compromising BBB. This review may provide a new angle to acknowledge the role of platelets in BBB integrity and vessel homeostasis.

## 4 Remaining questions and future directions

The role of platelets in BBB damage provides a new angle in exploring the pathological change of BBB under brain pathology. Even though a series of advancements have been achieved, the mechanism of platelet in BBB damage is still blur and needs further investigation. The AD aspects of the researches are relatively scattered but not systematic. To name a few, Aβ which can be released by platelets is commonly regarded as a hallmark molecule that leads to AD, one mechanism of which is to jeopardize BBB, but whether Aβ contributes to other nervous vascular disease such as ischemia stroke is not well known. In addition, accumulation of pTau, another leading cause of AD, of which role in BBB damage is still lacking of researches. Furthermore, the mechanism of above mentioned factors originated from the platelet needs further investigations of its role in BBB. For example, in contradiction with the role of PDGF-AA and PDGF-CC which were traditionally found to damage BBB, PDGFRα as their receptors were reported to protect BBB from one study. In addition, PNA is reported to secrete more than twice fold of ROS than neutrophils separately, which disturbs the function and integrity of BBB. However, the mechanism of the augmented effects by the interaction between platelets and neutrophils is still unknown. Moreover, PAF has been reported to mediate the aggregation between platelets and neutrophils, but whether it takes part in the formation of ROS is still unknown. The anti-platelet treatment may alleviate the secondary damage of cerebral hemorrhage and is maybe an important way to improve the prognosis of cerebral hemorrhage in patients. Nevertheless, further research to explore the balance between platelet activation and suppression is needed. At last, platelets transfusion has been regarded as a traditional therapeutic approach of hemorrhagic encephalopathy for a long period. However, previous studies also indicate that platelets can jeopardize BBB integrity. The question bewildered us would be how to keep the balance between the protective and the destructive effects on brain vasculature when applying platelets transfusion, of which the underlying mechanisms would be worth to explore.

## Author contributions

WL: Conceptualization, Writing – original draft, Writing – review & editing. XJ: Conceptualization, Funding acquisition, Supervision, Writing – review & editing. YZ: Conceptualization, Funding acquisition, Supervision, Writing – original draft, Writing – review & editing.
